# Supplementary material for: Machine Learning in Colorectal Cancer Risk Prediction from Routinely Collected Data: A Review
Source: Diagnostics (Basel). 2023 Jan 13;13(2):301. doi: 10.3390/diagnostics13020301 (PMC9858109; doi:10.3390/diagnostics13020301)
Supplement: Supplementary file 1 [file diagnostics-13-00301-s001.zip › diagnostics-2072557-supplementary.docx]

| Paper Authors | Data Source & Sample Size | Participants | Predictors & Outcome(s) | ML Development | Performance |
| --- | --- | --- | --- | --- | --- |
| Wan et al., 2019 | Single Institution registry (Japan).  ND - sample size not stated and number of cases not stated. | Consecutive - ND Incl/excl - yes include: tumor located in colorectal region, no contraindication to general anesthesia and surgery, and surgery occurred, pathology diagnosis after endoscopic surgery is intraepithelial neoplasia, clinical statistics are complete, informed consent and voluntary participation in clinical research are provided.  Exclude: clinical signs of colorectal intraepithelial neoplasia who showed metastasis, progressive CRC, taken anticoagulant drugs such as aspirin and clopidogrel within a week of surgery or patients with severe coagulopathy, severe cardiopulmonary dysfunction or patients at risk from other endoscopic treatments, pregnant or lactating women, partially or completely restricted consciousness and behavior as determined by ability. Additional criteria for removing: patients who have accidents during treatment process or need to undergo surgical treatment, patients who need additional surgery or other treatment methods after the endoscopic minimally invasive treatment, patients who have medical disputes. | 50 features assessed from endoscopic report, patient history and blood sample.  Outcome: Cancer risk (early stage) following colonoscopy. | Multiple ML models - SVM, KNN, ensembles for boosting and random forest with convolutional neural network, recurrent neural network and recursive neural network as deep learning. Selection limited by dataset Selection for model Inclusion in model determined by model type | Acc. 0.8148 Prec. 0.8571 Recall 0.6000 F-1 0.7059 (k=14) FNR 0.7321 |
| Wu H.-C et al., 2014 | Single Institution registry, not specified, (Taiwan, China).  343 initially considered, 225 used, 97 cases 150 train 75 test (45 normal and 32 cases?) | Consecutive - ND Symptomatic referred for colonoscopy Incl/excl - yes (nurse assessed) Exclusion criteria: 1. unable to answer questions from the investigator, 2. chart data incomplete, 3. failure to achieve complete colonoscopy without a diagnosis, 4. poor colon preparation (semisolid or solid stool at colonoscopy, 5.there was colorectal carcinoma with incomplete check of total colon | 20 features: demographic data (age, sex, body height, bodyweight, and body mass index), reasons for colonoscopy (bloody stool,abdominal pain, constipation, bowel habit change, anemia, tenes-mus, positive FOBT, colon polyp history, family history of CRC, orelevation in carcinoembryonic antigen level), and patient’s habits(smoking, drinking, betel nut chewing, or tea or coffee consumption).  Outcome: Adenomatous polyp risk. | Decision tree Variables from data set, 5 in final model (weight, age , height, BMI and sex) Selection using feature selection techniques and p-value cut-off "some normalisation" Simple, inetrmediate, and best models with different number of variables - selection related to 'complexity levels of parameters' but no details provided | Best performing decision tree had an AUROC of 0.937, accuracy 88%, specificity 92.2.%, sensitivity 82.5%, FPR 7.8% and FNR 17.5% (no confidence intervals reported for these measures. Plus support, confidence and odds provided for each leaf in tree. 5 rules LR as comparator (single rule): accuracy of 77.3%, FPR = 12.0%, FNR=24.7%, sens =75.3% , spec = 78.9%, AUC = 0.853. Confidence intervals not included . |
| Kinar et al., 2016 | Single institution registry (Israel) plus THIN from UK.  Table and text do not agree on figures, difference due to other cancers possibly. Supplementary materials not accessible to check. 466,107 (2437 cases) train 139,205 (698 cases) test 25,613 (5,061 cases) ext validation. | Consecutive - ND Incl/excl - yes - age > 40 in UK, diagnosed 2007-12, controls cancer free age 50-75 In Israel - insured individuals over the age of 40 2. In UK - individuals over age 40. All cancer cases in 2007-2012, then selected all CRC and random sample of cancer free controls who were aged 50-75 (reflects currrent guidelines for CRC screening in Israel). | Sex, birth year and blood counts (20 parameters).  Outcome: CRC prediction up to 2 years in advance. | Decision tree (gradient boosting model and random forest) Variables chosen in advance feature vector which includes age, sex, complete blood counts and trends (CBC at two time points) | AUROC Israel 0.82 (+/- 0.01) UK 0.81 (4 controls per case) OR, spec and sens reported. NO CIs for THIN validation set. Others in supplementary materials - not accessible. Specificity for 50% detection 87 +/- 2 (1 year pre diagnosis) |
| Hsieh et al., 2018 | Combined datasets incl. diabetes (subset of data of the National Health Insurcance Research Database (NHIRD) and the Longitudinal Cohort of Diabetes Patients (LHDP)) in Taiwan.  1,315,899 (97.5%) train 337,410 (2.5%) test (NB not 2.5%!) 14,867 cases, unclear how split between train and test. | Consecutive - ND Incl/excl - yes, include if two T2D diagnoses within the year (first diagnosis is index date), exclude if CRC prior to T2D, age < 20, incomplete demographic data | Demographic data, comorbidity and medication use 37 input features used. Age, Mean (SD) year, Gender, Urbanisation Level, Occupation, Hypertension, Hyperlipidemia, Stroke, Congestive Heart Failure, Colorectal polyps, Obesity, COPD, CAD, Asthma, Smoking, Inflammatory Bowel Disease, Irritable Bowel Syndrome, Alcohol related illness, CKD, Diabetes complication (aDCIs component), Retinopathy, Nephropathy, Neuropathy, Cerebrovascular, cardiovascular, Peripheral vascular disease, Metabolic, Mean aDCIs score Onset, End of follow up, Statins, Insulin, Sulfonylureas, Metformin, TZD, Other antidiabetic drugs and Mean follow up for endpoint. Selection of input features ND, the abstract states "All the available possible risk factors for CRC were also included in the analyses".  Outcome: CRC prediction. | Deep neural network, data normalisation Variable selection not described but as all 37 used in model selection within the neural network. An aggregated diabetes complication and severity index (score) was constructed as per (refs: Young 2008, Chang 2012) | AUROC test set 0.700 (0.674 - 0.727) Prec. 0.980 Recall 0.886 F-1 0.929  Compared with  all data & training data: f1 = 0.931, presision = 0.982, recall = 0.889, AUROC = 0.738 and 0.739 respectively, AUROC 95% CI = 0.734-0.742 & 0.735 - 0.743 respectively, AUROC SE = 0.002. test data:f1 =0.929, presicion = 0.980, recall = 0.886, AUROC = 0.700,AUROC 95% CI = 0.674-0.737 respectively AUROC SE = 0.013. |
| Wang Y.H. et al., 2019 | NHI dataset (Taiwan)  Five fold cross validation on 47,967 controls (from 50,000 randomly selected), 10,185 (all?) cases. | Consecutive - ND Incl/excl - yes CRC group: All patients with primary diagnosis of CRC a) they have ICD-9 codes nd codes 153, 154 AND b) have been included in inclusion in the Registry for Catastrophic Illness Patient (RCIP). Control group: randomly selected individuals without any cancer diagnosis during 15 years in the database.  Exclude if age < 20 years at the date of diagnosis CRC or no outpatient claims at each calendar year of four years before the index date | Comorbidity and medication use with age and sex 1931 features considered; age, sex, ICD9-CM codes (1099) and AT-C codes (830).  Outcome: CRC prediction within 1 year. | Neural network Variables as per data set, categorised into 19 groups Selected by model during run 2 convolutional layers with 32 feature maps for each category 2 max-pooling layers with the filters of size 1x3 were applied Age and sex added to flattened pooled features A hidden layer - 400 neurons was used in this fully-connected layer. Normalisation was performed on data, 1929 features grouped to 19 | AUROC 0.922 (SD 0.004) Sens. 0.837  Spec. 0.867 PPV 0.532 |
| Hornbrook et al., 2017 | Single Institution registry (US) - Kaiser Permanente Northwest Region.  17,095 eligible (900 cases, 9108 controls) | Consecutive - ND Incl/excl - yes - CRC with CBC pre diagnosis, random controls with no cancers, with a CBC, data for 180 days pre CBC and 2 years after, aged 40 - 89 Pseudo-CRC diagnosis date for matching, 18 controls per case diagnosis year | Gender, age and blood count (number of parameters in CBC unclear "at least one").  Outcome: Prediction of CRC detection within 6 months to 1 year | Decision tree (gradient boosting model and random forest) Variables chosen in advance feature vector which includes age, sex, complete blood counts and trends (CBC at two time points) | AUCROC 0.8 (0.79 - 0.82) OR at 99% specificity 34.7 (28.9 - 40.4) Odds ratios reported and sensitivity and specificity reported for age groups and time windows prior to diagnosis The detection model had the highest accuracy in identifying right-sided colorectal cancers. |
| Birks et al., 2017 | Clinical Practice Research Datalink (CPRD) (UK).  2,914,589 eligible, 2,550,119 after exclusions, 25,430 (~ 1%) cancers (events) Another 68 excluded?? diagnosis < 40 years 2,220,108 controls and 5,141 cases in primary analysis. | Consecutive - ND Incl/excl - yes - at lease one FBC, age > 40 years. Exclude if registered with GP practice less than 12 months, less than 2 years of follow up data, history of CRC, had CRC precursors, had Hb gene defects. | Gender, age and blood count (20 parameters).  Outcome: CRC prediction in 18 - 24 months diagnosed in primary care. | Decision tree (gradient boosting model and random forest) Variables chosen in advance feature vector which includes age, sex, complete blood counts and trends (CBC at two time points) Original paper reported: Selecting model features: created a feature vector which includes age, sex, complete blood counts and trends (CBC at two time points). Evaluation of the vector is using linear regression. Classification method: Used decision trees. They used 2 methods - the Gradient Boosting Model and random forests. Used Area under the curve for measuring performance. | AUROC 18-24 months 0.776 (0.771 – 0.781) Spec. 82.73 (82.68 – 82.78) Sens. 3.91 (3.40 – 4.48) PPV 8.8% NPV 99.6% |
| Kinar et al., 2017 | Health care insuranceprovider (MHS) and cancer Registry (Israel).  112,584 with 133 cases NB Used scores. | Consecutive - ND Incl/excl - yes - age 50-75 years on Jan 1st 2008 with one or more complete blood count reports recorded (MHS) between 1st July 2007 and 31 Dec 2007. Excusion if diagnosis of CRC or any cancer prior to 1st Jan 2008, no complete blood count during the 6 month testing period. (where > 1 CBC taken the last one was taken as the index test). | Gender, age and blood count (20 parameters) at 2 time points.  Outcome: CRC prediction within 6 months to 1 year | Classification, decision trees. Combined the Gradient Boosting Model and random forest. Final prediction is a score based on the two models. | Reported sensitivity, yield and odds ratio at 1% and 3% positive rate cut offs: 1%  sensitivity 17.3% Yield 2.1% Odds ratio: 21.8 (13.8 - 34.2) diag in 6 months: sensitivity 25% diag 7 - 12 months sensitivity 9.5% 3% cut off sensitivity 24.8% Yield 1% Odds ratio: 10.9 (7.3 - 16.2) diag in 6 months: sensitivity 29% diag 7 - 12 months: sensitivity 20% |
| Goshen et al., 2017 | Maccabi Health Services and Registry (Israel)  102,775 controls and 2294 cases, 1755 cases and 54,730 iteratively matched controls used in study. | Consecutive - ND Incl/excl - yes - include cases (CRC) diagnosed between 2002 and 2011 aged 40 - 75 and at least one blood count pre diagnosis - exclude if CRC/other cancer before 2002. 45 controls per case with one blood count pre match index case - matched on sex and year of birth. | Gender, age, blood count , liver function , metabolic blood tests and iron, folic acid and vitamin B12 levels - table 2 - 30 considered.  Outcome: CRC prediction within 6 months to 2-3 years. | Feature selection by logistic regression, cut off p < 0.0001 and OR > 1.5 (different values / features selected in males v females) - 9 used in final model Final model also logisitic regression | Scoring model with a score of >= 4 as cut off level had an odds ratio (versus < 4) of 7.3 (6.3 - 8.5) for men and 7.8 (6.7 - 9.1) for women. Predicts risk 30 - 180 days pre diagnosis. High risk score: males 4+ score Sensitivity 31% Specificity 95% PPV of abnormal score 7.3% females 5+ score sesnsitivity 24% specificity 95% PPV 4.2% |
| Hilsden et al., 2018 | Single Institution registry (Canada) Alberta Health Services’ Forzani & MacPhail Colon Cancer Screening Centre in Canada. Linked medical records and laboratory data.  17,676 of which there were 60 CRC cases and 1,104 high risk polyps. | Consecutive - ND Incl/excl - yes - aged 50 to 75 underwent colonoscopy at the Centre between January 2013 and June 2015, undergone a successful colonoscopy (complete to the cecum unless incomplete due to an obstructing mass) with a bowel preparation rated by the endoscopist as adequate to detect polyps greater than 5 mm in size. Three subgroups of patients were eligible for the study; individuals at average risk for colorectal cancer, individuals with a personal history of polyps and individuals with a family history of polyps or colorectal cancer. Exclusion if they had a positive guaiac or immunochemical fecal occult blood test, prior history of colorectal cancer, known or suspected genetic predisposition to cancer or no CBC result within the year prior to colonoscopy. | Gender, age and blood count (1 or more from Hb, haematocrit, MCV, MCH, RBC, red blood cell ditribution width, WBC, platelets, % neutrophils, lymphocytes, monocytes, eosinophils and basophils). (up to 15 variables).  Outcomes: Prediction of CRC and high risk pre-cancerous lesions. | Using the Kinar developed algorithm - combined a Gradient Boosting Model and Random forest | OR for positive result was 5.1 (2.3 - 8.9) at 95% specificity for CRC with an OR of 2 (1.6 - 2.6) for advanced precancerous lesion, OR 1.7 (1.5 - 2.0)for non-advanced polyp versus no findings at colonoscopy. Breakdown for subgroups also report sensitivity with CIs e.g. all patients: sensitivity 8.1 (6.4 - 9.8) at 95% specificity. |
| Kop et al., 2016 | GP EMR dataset (Netherlands) Julius General Practitioners’ Network, Utrecht; Academic Network of General Practice VU University Medical Center Amsterdam (ANH VUmc); Leiden General Practitioner Registration Network RNUH-LEO, LUMC, Leiden.  263,879 of which 1292 are cases. | Consecutive - ND Incl/excl - yes - age >= 30, at least 6 months EMR data prior to CRC (random 6 months if no CRC) - exclude if < 6 months Patient selection - Patient invited for FIT screening. Detailed description in previous study. Inclusion: a) participants aged 60 to 74 years old b) participants with complete data records, c) FIT result > = 20mcg/g -1 d) a definitive colonoscopy outcome. Individuals with a positive FIT test were offered a specialist screening practitioner appointment within 14 days of FIT test and, if appropriate, referred for a colonoscopy within 14 days of the appointment. | Age and gender with medical record based data for consultations, medication, referral, diagnoses and lab test results: drugs for constipation; iron deficiency anemia; lipid modifying agents (s) ; drugs for constipation; age; drugs for acid related disorders (s) ; drugs for constipation; diabetes non-insulin dependent; abdominal pain/cramps general; diabetes non-insulin dependent (s); diabetes non-insulin dependent; beta blocking agents (s) ; drugs for constipation; hypertension uncomplicated (s) ; hypertension uncomplicated; agents acting on the renin-angiotensin system (s); drugs for constipation; diuretics; flu vaccination (a); agents acting on the renin-angiotensin system (s); Antithrombotic agents; abdominal pain localized other; general consult (s) general consult (s) general consult; agents acting on the renin-angiotensin system (s) ; drugs for acid-related disorders; agents acting on the renin-angiotensin system.  Outcome: Occurrence of CRC. | Multiple as part of a pipeline - CART, LR and RF; LR performed best. Variables from dataset identified from temporal patterns. | LR: Data-driven AUROC 0.891 Prec. 0.03 Recall 0.642 F1 score 0.058 Same info reported for A&G, BB, CART and RF (all poorer than LR) A&G AUROC 0.836 Precision 0.022 Recall 0.455 F1 score 0.041 BBE AUROC 0.864 Precision 0.026 Recall 0.537 F1 score 0.049 |
| Cooper et al., 2018 | Screening Hub Data (UK) Two regional screening hubs in England: (i) the Midlands and North West Hub and (ii) the Southern Hub.  1810 for complete case analysis of which 72 cancers 214 high risk adenomas 262 intermed risk ademonas (cases) 468 low risk (negative) NB Inconsistencies between study flow diagram and results section. | Consecutive - ND Incl/excl - yes - FIT screening invite (detailed description in previous study) Include: age 60 to 74 years old, complete data records, FIT result > = 20mcg/g, definitive colonoscopy outcome. Positive FIT test offered specialist screening practitioner appointment within 14 days of FIT test and, if appropriate, referred for colonoscopy within 14 days of the appointment. | Age, sex, deprivation index, screening history, FIT test result.  Outcomes: Risk prediction, including FIT result, for subsequent colonoscopy within 14 days. | LR and neural network Two LR models: FIT concentration only and Risk adjusted model - FIT concentration and routine data Risk-adjusted model backwards elimination method was used to remove nonsignificant variables (p>0.1), 10 fold cross-validation to address overfitting.  Sample partitioning for cross-validation unclear. Multilayer ANN model had an input layer (with same predictors from LR model), a single hidden layer and an output layer with a single node. Networks pruned by dropping out weights with lowest magnitude and assess change in cross-validated deviance Variables as per dataset Selection for inclusion as per prev. studies Assessed by backward elimination by multivariate analysis and cutoff for EXCLUSION p > 0.1 | ANN: AUROC 0.686 (0.659 – 0.712) Sens. 35.15% Spec. 85.57% PPV 51.47 NPV 75.19 FIT +ve 20.72% CRC detection 10.66%  FIT only AUROC 0.628 (0.600 - 0.656) Sens. 30.78% Spec. 83.66% PPV 45.07% NPV 73.52% FIT +ve 20.72% CRC detection 9.34%  Risk adjusted: AUROC: 0.659 (0.632 - 0.686) Sens. 33.15% Spec. 84.69% PPV 48.53% NPV 74.42% FIT +ve 20.72% CRC detection 10.6% |
| Nartowt B. J. et al., 2019 | NHIS Questionnaire (USA).  525,394 train (1,269 cases) 58,376 test (140 cases). | Consecutive - ND Incl/excl - yes - 20% excluded for missing data, - exclude if CRC diagnosis > 4 years after survey response (NB age range unclear, > 18 years) | Health questionnaire responses: Current or cancer age, hypertension, number of first-degree relatives with CRC (NHIS years 2000, 2005, 2010, and 2015 only), coronary heart disease, pooled heart conditions, myocardial infarction, diabetes (non-gestational), heart condition/disease , vigorous exercise frequency, angina pectoris, ulcer (stomach, duodenal, peptic), Hispanic ethnicity, stroke, emphysema, American Indian, African American, other, or multiple race, sex (male), body-mass index, smoking frequency.  Outcome: CRC risk for screening, within 4 years. | Neural network, four layers with 12 to 14 inputs, each hidden layer with same number of neurons as input layer Variables chosen from dataset Inclusion if previous correlation with CRC Pearson correlation for variables Ten-fold cross-testing and back-propogation | Performance presented in multiple ways AUCROC 0.80 (+/- 0.05) - concordance Sens. 0.57 (+/- 0.03) Spec. 0.89 (+/- 0.02) PPV 0.0075 (+/- 0.0003) - value only in abtsract NPV 0.999 (+/- 0.001) - value only in abstract |
| Shi et al., 2019 | Study data were from two independent hospitals in Beijing, China: Peking University Cancer Hospital (PUCH) and Peking University Shougang Hospital (PUSH). Specialist routine lab tests for cases and physical health examination centres for controls.  PUCH data set consists of 7,068 diagnosed CRC cases from 2010 to 2015 and 80,194 controls who received physical health examinations but were not clinically diagnosed with CRC from 2007 to 2014. The PUSH data set consists of 453 CRC cases from 2011 to 2016 and 66,570 controls from 2009 to 2016. After data QC, 4,211 cases and 77,099 controls in PUCH and 436 cases / 56,799 controls in PUSH. PUSH - Training / test split was 80/20 with training set further split 75/25 for model development. PUCH - 70/30 training/test split, validated on the PUSH 30%. | Consecutive - ND Incl/excl - ND well, received physical examination or CRC diagnosis (with tests) | variables selected during model process as refinement of CART. From these which were included on basis of least missing values:  A/G, rate of albumin to globulin; Alb, albumin; ALT, alanine transaminase; AST, aspartate transaminase; BASO%, percent basophils; Ca, calcium; Crea, creatinine; EO%, percent eosinophils; Glu, glucose; HCT, hematocrit; HDL-C, high density lipoprotein-cholesterol; HGB, hemoglobin; K, kalium; LDL-C, low density lipoprotein-cholesterol; LYMPH%, percent lymphocytes; MCH, mean corpuscular hemoglobin; MCHC, mean corpuscular hemoglobin concentration; MCV, mean corpuscular volume; MONO%, percent monocytes; MPV, mean platelet volume; NEUT%, percent neutrophils; P, phosphorus; P-LCR, platelet large cell ratio; PCT, plateletcrit; PDW, platelet distribution width; PLT, platelet; RBC, red blood count; RDW-CV, variable coefficient of red blood cell distribution width; RDW-SD, standard deviation of red blood cell distribution width; TBil, total bilirubin; TCHO, total cholesterol; TG, triglyceride; TP, total protein; UA, uric acid; WBC, white blood count Final model used: age, albumin, haematocrit, % lymphocytes.  Outcome: Colorectal cancer risk prediction, timeframe from diagnosis unclear. | Classification and regression tree. | AUC of the CART model on PUCH internal validation was 0.88 (95% CI, 0.87−0.90) and the sensitivity was 62.2% (95% CI, 58.1%−66.2%) at the 99.0% specificity. From development test set:  AUC values of the CART and age models were 0.90 (95% CI 0.88−0.91) and 0.81 (95% CI, 0.80−0.82), respectively. Sensitivity of the CART model was 67.0% (95% CI, 63.7%−70.2%), much higher than 4.8% (95% CI, 3.2%−6.4%) of the age model when defining cutoffs yielding 99.0% specificity CART model can correctly identify 67% of real CRC cases at the cost of misclassifying 1% of CRC-free individuals as CRC cases |
